# Effects of Neuromuscular Electrical Stimulation of Gluteus Medius Muscle on Hip Function and Sleep Quality in Patients With Sleep Disorders After Hip Arthroplasty

**DOI:** 10.62641/aep.v54i3.2248

**Published:** 2026-06-15

**Authors:** Yang Zhou, Yanmei Shen, Xianping Lin, Chaohui Huang, Jie Sun, Zizheng Wu, Liang Lu, Chunxia Qian, Ting Zhang

**Affiliations:** ^1^Department of Orthopedic Rehabilitation II, The First Rehabilitation Hospital of Shanghai, 200090 Shanghai, China; ^2^Department of General Practice, Shanghai Yangpu District Daqiao Community Heath Care Center, 200090 Shanghai, China; ^3^Department of Critical Care Rehabilitation for Respiratory Disorders, The First Rehabilitation Hospital of Shanghai, 200090 Shanghai, China; ^4^Department of Rehabilitation Medicine, Shanghai Baoshan District Wusong Central Hospital (Zhongshan Hospital Wusong Branch, Fudan University), 200940 Shanghai, China

**Keywords:** neuromuscular electrical stimulation, gluteus medius, hip arthroplasty, sleep disorder, pain, hip function

## Abstract

**Background:**

This study aimed to investigate the effects of neuromuscular electrical stimulation (NMES) of gluteus medius on hip function and sleep quality in patients with sleep disorders after hip arthroplasty (HA).

**Methods:**

This retrospective analysis included 100 patients who underwent HA between January 2022 and August 2025 and developed postoperative sleep disorders. Patients were divided into in a control group (n = 45) and an NMES group (n = 55) based on the treatment received. The NMES group received NMES applied to the gluteus medius; both groups were treated for 4 weeks. Patient data extracted from the medical record system included hip function scores and sleep records: visual analogue scale (VAS), Harris Hip score, Berg Balance Scale (Berg) score, and Pittsburgh Sleep Quality Index (PSQI) score. These measures were compared between the two groups before and after treatment. Therefore, the correlation between VAS score and PSQI score was analysed.

**Results:**

After treatment, hip function and sleep quality improved in both groups. VAS scores decreased significantly after 4 weeks of treatment in both groups (*p *< 0.05), with the NMES group showing lower scores than the control group (*p *< 0.05), Harris and Berg scores increased significantly in both groups (*p *< 0.05), and both were higher in the NMES group (*p *< 0.05). The PSQI score in the NMES group was lower than that in the control group (*p* < 0.05). Correlation analysis showed a strongly positively correlated between VAS and PSQI scores in both groups before treatment (*p* < 0.05); after treatment, a weak positive correlation remained in the NMES group, whereas a moderate positive correlation persisted in the control group.

**Conclusions:**

Applying NMES to the gluteus medius is associated with reduced pain intensity in patients with sleep disorders after HA. This may contribute to improved sleep quality and help alleviate the reciprocal interaction between pain and sleep disorders.

## Introduction

The number of hip arthroplasty (HA) procedures in China has increased substantially over the past decade, more than doubling between 2011 and 2019 [1]. This trend is expected to continue over the next 10 years [2]. HA is the gold standard for treating end-stage hip diseases (such as femoral head necrosis, hip osteoarthritis, femoral neck fracture), as it effectively restores hip function, relieve joint pain, and improve patients’ quality of life [3]. However, many postoperative problems persist, including hip muscle atrophy, impaired balance, reduced walking ability and postoperative pain, all of which can easily induce sleep disorders [4]. A study reported a 55.3% incidence of sleep disturbance on the first postoperative day, primarily manifested as difficulty falling asleep, shallow sleep, frequent nocturnal awakening and low sleep efficiency [5]. Sleep disorders not only reduce physical and mental well-being of patients, but also disrupt the neuro-endocrine-immune network, inhibit tissue repair and bone healing, reduce adherence to rehabilitation training, and consequently delay the hip function recovery. This creates a vicious circle of “pain-sleep disturbance-delayed functional recovery”, which seriously affects the postoperative rehabilitation process and prognosis [6].

Postoperative problems after HA are closely related to the stability of the posterior hip muscle group, with the gluteus medius playing a particularly prominent role [7]. The gluteus medius often develops muscle weakness and dysfunction due to surgical trauma, braking and other factors, leading to hip biomechanical imbalance, which further aggravates particular tissues traction and pain. This is also an important indirect factor in inducing and worsening postoperative sleep disorders [8]. Concurrently, intraoperative muscle dissection and postoperative temporary never inhibition cause more pronounced damaged to the gluteus medius after HA. Thus, precision rehabilitation of gluteus medius is critically important for patients after HA.

Dysfunction of the gluteus medius, a core stabilizing muscle group around the hip joint, is major cause of hip instability and exacerbated pain after HA. Neuromuscular electrical stimulation (NMES) uses low-frequency pulsed currents to simulate muscle contraction, promote local blood circulation, improve muscle function, and relieve pain [9, 10]. In recent years, NMES has gained attention for rehabilitation after HA, as it can activate muscles, increase muscle strength, and alter muscle morphology [11, 12, 13]. However, current application of NMES after HA primarily focuses on hip function recovery, with limited discussion of its effect on sleep quality in patients with postoperative sleep disorders or the “pain relief-sleep improvement” mechanism. Although NMES is increasingly used in orthopaedic postoperative rehabilitation, studies specifically targeting improvements in hip function and sleep quality in HA patients with postoperative sleep disorders remain scarce. As a non-invasive physical therapy, NMES can precisely target specific muscles and has a multidimensional potential mechanism for improving sleep disorders when applying to the gluteus medius: promoting local blood circulation, clearing pain-causing substances, relieving muscle spasms via electrical stimulation, thereby directly reducing nocturnal pain interference with sleep; improving gluteus medius function to enhance in bed comfort and reducing the pain stress from positional changes; and regulating central neurotransmitters through peripheral afferent nerves, inhibiting pain sensitization, balancing sleep-wake rhythms, and suppressing sympathetic nerve overactivation, thereby reducing the overall stress response and creating a favourable physiological environment for sleep. This study investigated the effects of gluteus medius NMES on sleep quality and hip function in 100 patients with sleep disorders after HA, focusing on the pain-relief pathway to improve sleep, with the aim of providing evidence-based support for optimizing postoperative rehabilitation protocols and improving of patient outcomes.

## Materials and Methods

### Baseline Data

A retrospective analysis was conducted on the case data of 100 patients with sleep disorders after HA who were hospitalized in Shanghai Baoshan District Wusong Central Hospital and The First Rehabilitation Hospital of Shanghai from January 2022 to August 2025. Patients were divided into a control group and an NMES group based on actual clinical postoperative rehabilitation strategies. The assignment was determined by the attending physicians according to the patients’ rehabilitation willingness, functional status, and routine clinical practice, rather than random allocation.

### Inclusion and Exclusion Criteria

#### Inclusion Criteria

(1) Meet the indications for HA and successfully underwent the procedure; (2) diagnosis of sleep disorders based on the Diagnostic and Statistical Manual of Mental Disorders (Fifth Edition) (DSM-5) criteria [14], with postoperative Pittsburgh Sleep Quality Index (PSQI) score of > 7 (PSQI > 7 indicates clinical diagnosed sleep disorders) [15]; (3) with normal cognitive function, patient’s scale results were obtained from laboratory records; (4) age ≥ 18 years; and (5) complete and clear case data.

#### Exclusion Criteria

(1) Severe cardiovascular or cerebrovascular diseases, liver and kidney insufficiency, malignancy or other underlying diseases; (2) neurological diseases or mental illnesses (such as acute episodes of depression or anxiety); (3) contraindications to NMES (such as implanted pacemaker, defibrillator, skin breakdown, infection or coagulation dysfunction) at the treatment site; (4) serious postoperative complications (such as prosthesis dislocation, incision infection, deep vein thrombosis); (5) history of chronic sleep disorders (persistent sleep disorders within 6 months before surgery); and (6) concurrent treatment with other therapies that could affect the study outcomes.

#### American Society of Anaesthesiologists Physical Status Classification

The American Society of Anaesthesiologists (ASA) classification has six grades. ASA I: A normal healthy patient, ASA II: A patient with mild systemic disease, ASA III: A patient with severe systemic disease, ASA IV: A patient with severe systemic disease that is a constant threat to life, ASA V: A moribund patient not expected to survive without the operation, ASA VI: A declared brain-dead patient whose organs are being removed for donation [16].

This study was approved by the Ethics Committee of Shanghai Baoshan District Wusong Central Hospital (Zhongshan Hospital Wusong Branch, Fudan University) (Ethics Number: 2026-P-01) and the Ethics Committee of The First Rehabilitation Hospital of Shanghai (Ethics Batch Number: YK-2025-03-012) and was conducted in accordance with the principles of the Declaration of Helsinki. All eligible participants signed an informed consent form.

### Treatment Methods

Case data were collected from two hospitals. To ensure the uniformity of case treatment, we evaluated the treatment protocol and standard operating procedures of both hospitals to confirm that the treatment content, implementation steps, duration, and frequency were consistent. Designated personnel supervised the implementation to avoid bias from procedural differences. Additionally, we verified to ensure that the instruments, scales, and environmental conditions used for assessments and treatments remained uniform across sites. Both groups received basic drug therapy and conventional rehabilitation treatment for 4 weeks.

#### Basic Treatment

Postoperative routine anti-infective and basic drug treatment, including antihypertensive, hypoglycaemic, lipid-lowering, anticoagulant, nutritional support and other related medications, but excluding anti-inflammatory and analgesic drugs. Concurrently, patient received nutritional support and psychological counselling and were guided to develop regular sleep-wake cycle and avoid strenuous activities and emotional fluctuations before bedtime.

Routine rehabilitation training: supervised by professional rehabilitation therapists, including: (1) 1–2 weeks after surgery: ankle pump exercises, isometric quadriceps contraction training, passive hip flexion and extension training (angle control at 0°–90°) to prevent deep vein thrombosis and muscle atrophy; (2) 3–4 weeks after surgery: active hip flexion and extension, abduction training, assisted walking training (with the help of walkers), and balance function training to gradually restore hip mobility and limb coordination. Training intensity was adjusted according to patient tolerance, 30 minutes per sessions, once a day, and 5 days per week.

#### Electrical Stimulation of the Gluteus Medius Nerve Muscular Stimulation

The NMES group additionally received NMES applied to the gluteus medius on the affected side. The treatment was delivered for 30 minutes a day, once a day, for 4 consecutive weeks. NMES parameters were set as follows: pulse width 300 μs, bidirectional wave, output frequency 40 Hz, current intensity range 0–120 mA, power supplied by a constant voltage system approximately 150 V. Stimulation intensity was gradually adjusted according to patient tolerance, with 10 seconds on and 20 seconds off, the current research method is adapted from the cited studies. The parameters used in this study fall within the widely accepted therapeutic range of NMES reported in previous literature [17]. Electrode pad placement: All procedures were performed by the same experienced rehabilitation therapist. The stimulation target point for the gluteus medius was identified along the line from the anterior superior iliac spine to the outer upper one third of the coccyx. The monitoring electrode was placed at the marked point on the affected hip, and the other electrode was placed over exit of the gluteal epithelial nerve on the affected side. During stimulation, patients in the NMES group were asked to voluntarily contract the gluteus medius [18].

### Hip Joint Function Test

Patient records of visual analogue scale (VAS), Harris score, and Berg Balance Scale (Berg) at week 0 and week 4 were reviewed to obtain these scales results.

#### VAS Score

The VAS is a simple and intuitive subjective pain assessment tool widely used for quantitative assessment of various clinical pains (such as postoperative pain, chronic pain, cancer pain), particular in adult patients who can normally express subjective feelings [19]. Patients mark their subjective pain level on a 10 cm linear scale: 0 points = no pain; 1–3 points = mild discomfort; 4–6 points = moderate discomfort; 7–10 points = severe discomfort. The test–retest correlation coefficient [ICC (intra-group correlation coefficient)/Pearson correlation coefficient] of VAS score is 0.85.

#### Harris Score

The Harris score scale was proposed by American orthopaedic surgeon William Harris in 1969 and has been widely used internationally for hip function assessment [20], which covers four dimensions: pain, function, joint deformity and mobility, with a score of 0–100 points. Harris score is convenient to administer and has high reliability and validity. Scoring criteria: 90–100 = excellent, 80–89 = good, 70–79 = acceptable, <70 = poor, which are used for postoperative and functional evaluation of hip replacement, hip fracture, osteoarthritis [21]. Cronbach’s α coefficient for the Chinese version is 0.811, and the Slovenian version is 0.94.

#### Berg Score

The Berg was developed by Katherine Berg in 1989 and is one of the most commonly used clinical tools for assessing balance function [22]. The scale contains 14 items, each scored from 0 to 4 (4 = best completion, 0 = unable complete), for a total score of the scale is 0–56 points. Higher scores indicate better balance and lower fall risk. The Cronbach’s α coefficient of the Berg is 0.91, indicating excellent internal consistency. The test–retest reliability shows an ICC of 0.88 (95% confidence interval [CI]: 0.80–0.93), indicating good reliability.

### Sleep Quality Data

PSQI is a standardized self-rate scale that evaluates sleep quality over the past month, and comprehensively reflects both subjective and objective sleep dimensions, and is suitable for screening and severity assessment of sleep disorders. The Chinese version of the PSQI revised by Liu *et al*. [23] includes 7 dimensions (such as subjective sleep quality, sleep latency, sleep duration, sleep efficiency, sleep disturbances, use of hypnotic medication and daytime dysfunction), consisting of 19 self-rated questions and 5 questions rated by sleep partners, and only 19 self-rated questions are scored. Each dimension is scored on a 0 to 3 scale (0 points = none, 1 point = mild, 2 points = moderate, 3 points = severe), and the 7 dimensions scores are summed for a total score (0–21). A total score of > 7 suggests a sleep disorder [24]. The Chinese version of the PSQI has a Cronbach’s α coefficient of 0.83 and a test–retest reliability of 0.82, indicating good reliability and validity.

Sleep quality data were selected for analysis before treatment and 4 weeks after treatment.

### Sleep Duration

Sleep duration followed the unified definition from the International Classification of Sleep Disorders, 3rd edition (ICSD-3) and the sleep health standards for Chinese adults [25]. Nighttime sleep duration was defined as the actual sleep duration between 22:00 and 08:00 the next day. Subjects were continuously monitored objectively for 7 consecutive days using a portable intelligent sleep monitoring wristband (Model: WS20A) to further verify the accuracy of the subjective measurement. The monitor recorded the sleep-wake cycles via sensors, automatically identifying sleep onset time, wake-up time and sleep duration. After data export, professional personnel checked and corrected the data, excluding abnormal data caused by equipment malfunctions or improper wearing and other factors. During the monitoring period, subjects were required to maintain a regular daily schedule and avoid sleep-affecting behaviours such as alcohol consumption and sedative-hypnotic drug use to ensure data authenticity. Adults aged 18 to 64 require 7 to 9 hours of normal sleep duration. Less than 7 hours or more than 9 hours both constitute abnormal sleep health [25].

### Statistical Analysis

SPSS (Version 26.0; IBM Corp., Armonk, NY, USA) was used for data analysis. Normality of measurement data was assessed using the Shapiro-Wilk test. Normal distributed data are presented as mean ± standard deviation, non‑normally distributed data as median (interquartile range). Homogeneity of variance between groups was evaluated using Levene’s test. Paired t-tests were used for within group comparison before and after treatment, and independent sample t-test for comparison between groups comparisons. The interaction effect of multiple time points and between groups was analysed using repeated measures ANOVA. Categorical data are expressed as rates (%) and analysed, using the χ^2^ test. Pearson correlation analysis was used to examine the correlation between VAS score and PSQI score before treatment and after 4 weeks of treatment; the correlation coefficient r indicated the strength of association. A *p* < 0.05 was considered statistically significant.

## Results

### General Information

Based on the different treatment received, patients were divided into a control group (n = 45 cases) and an NMES group (n = 55 cases). No significant differences were observed between the two groups in gender, age, aetiology, PSQI score, body mass index and other general data (*p* > 0.05), indicating comparability (Table 1).

**Table 1.** **General information of patients**.

Indicator	Control group (n = 45)	NMES group (n = 55)	t/ χ²	*p*
Gender (male/female)	17/28	22/33	0.051	0.821
Age	76.35 ± 5.24	75.12 ± 5.74	1.108	0.270
Cause (femoral head necrosis/hip arthritis)	38/7	46/9	0.012	0.913
BMI	24.68 ± 2.18	25.01 ± 2.34	0.723	0.471
PSQI score	15.62 ± 4.25	14.94 ± 3.87	0.816	0.416
ASA PS Classification (ASA I/ASA II/ASA III/ASA IV)	10/11/18/6	12/16/20/7	0.293	0.961
Type of surgery (Total hip arthroplasty/ Hemihip replacement)	31/14	37/18	0.030	0.863
Smoking	32 (71.11)	38 (69.09)	0.048	0.826
Alcohol consumption	23 (51.11)	29 (52.73)	0.026	0.872

Note: NMES, neuromuscular electrical stimulation; BMI, body mass index; PSQI, Pittsburgh Sleep Quality Index; ASA, American Society of Anaesthesiologists; PS, physical tatus.

### Comparison of VAS Score and Harris Score Before and After Treatment

Before treatment, no significant difference was observed in VAS score and Harris score between the two groups (*p* > 0.05). After 4 weeks of treatment, VAS score decreased significantly from baseline in both groups, and Harris score increased significantly from baseline, with statistically significant differences (*p* < 0.05). The NMES group had lower VAS score, and higher Harris score than the control group, with statistically significant between group differences (*p* < 0.05, Table 2).

**Table 2.** **VAS score and Harris score before and after treatment**.

Groups	n	VAS score		Harris score	
		Before treatment	After treatment	Before treatment	After treatment
Control group	45	6.85 ± 1.28	3.54 ± 0.93*	52.34 ± 6.52	75.61 ± 7.22*
NMES group	55	6.93 ± 1.34	2.18 ± 0.74*	53.12 ± 6.81	86.41 ± 7.51*
*t*		0.303	8.145	0.629	6.747
*p*		0.763	<0.001	0.531	<0.001

Note: NMES, neuromuscular electrical stimulation; VAS, visual analogue scale. Intra-group comparison before and after treatment, *p < 0.05.

### Comparison of Berg Scores Before and After Treatment

Before treatment, no significant difference was observed in Berg score between the two groups (*p* > 0.05). After 4 weeks of treatment, Berg score increased significantly from baseline in both groups (*p* < 0.05). NMES group had higher Berg scores than the control group, with a statistically significant between group difference (*p* < 0.05, Table 3).

**Table 3.** **Comparison of Berg scores before and after treatment**.

Groups	n	Berg score	
		Before treatment	After treatment
Control group	45	32.52 ± 4.35	41.23 ± 4.93*
NMES group	55	33.12 ± 4.86	45.78 ± 5.74*
*t*		0.644	4.199
*p*		0.521	<0.001

Note: NMES, neuromuscular electrical stimulation; Berg, Berg Balance Scale. Intra-group comparison before and after treatment, *p < 0.05.

### Comparison of PSQI Scores and Nighttime Sleep Duration Before and After Treatment

Before treatment, no significant difference was observed in PSQI scores between the two groups (*p* > 0.05). After 4 weeks of treatment, PSQI score decreased significantly from baseline in both groups, and nighttime sleep duration increased significantly (*p* < 0.05). After 4 weeks, the NMES group had lower PSQI score and longer sleep duration than the control group, with statistically significant between group differences (*p* < 0.05, Table 4).

**Table 4.** **Comparison of PSQI score and nighttime sleep duration before and after treatment**.

Groups	n	PSQI score		Nighttime sleep duration	
		Before treatment	After treatment	Before treatment	After treatment
Control group	45	15.62 ± 4.25	7.88 ± 1.25*	5.56 ± 1.28	6.33 ± 2.64*
NMES group	55	14.94 ± 3.87	6.25 ± 1.33*	5.32 ± 1.23	7.10 ± 2.35*
*t*		0.816	6.263	0.953	2.576
*p*		0.416	<0.001	0.343	<0.05

Note: PSQI, Pittsburgh Sleep Quality Index; NMES, neuromuscular electrical stimulation. Intra-group comparison before and after treatment, *p < 0.05.

### Correlation Analysis Between VAS Score and PSQI Score Before and After Treatment

Before treatment, VAS score and PSQI score were strongly correlated in both the control and NMES groups (*p* < 0.05), indicating that more postoperative pain was associated with worse sleep quality. After 4 weeks of treatment, VAS and PSQI score remained positively correlated in both groups, but the correlation coefficients were significantly lower than before treatment. The decrease was more significant in the NMES group, and the difference in correlation strength between groups was statistically significant (*p* < 0.05). The control group showed a moderate positive correlation after treatment, whereas NMES group showed a weak positive correlation. (Table 5).

**Table 5.** **Correlation analysis between VAS score and PSQI score before and after treatment**.

Groups	n	Time	Correlation coefficient (r/ρ)	95% CI	*p*-value	Degree of association
Control group	45	Before treatment	0.752	0.590–0.854	<0.001	Strong positive correlation
		After treatment	0.426	0.158–0.634	0.003	Moderate positive correlation
NMES group	55	Before treatment	0.761	0.623–0.853	<0.001	Strong positive correlation
		After treatment	0.283	0.026, 0.503	<0.05	Weak positive correlation

Note: VAS, visual analogue scale; PSQI, Pittsburgh Sleep Quality Index; CI, confidence interval; NMES, neuromuscular electrical stimulation. The absolute value of the correlation coefficient is 0.00–0.19 is very weak correlation, 0.20–0.39 is weak correlation, 0.40–0.69 is moderate correlation, and 0.70–1.00 is strong correlation.

## Discussion

The incidence of sleep disorders after HA is high, and the pathogenesis is complex, primarily related to postoperative pain, postural discomfort caused by limited hip function, psychological stress, and neurological dysfunction [26]. As the main source of postoperative stress, pain activates sympathetic nervous system through neural transmission, leading to increased heart rate and blood pressure, thereby disrupting sleep rhythm [27]. Concurrently, postoperative gluteus medius dysfunction reduces in hip joint stability, further affecting patient mobility and position changes, exacerbating sleep disorders, and delaying the recovery. Therefore, relieving pain, improving gluteus medius function, and breaking the “pain-sleep disorder” cycle are key to promoting recovery.

NMES is a non-invasive rehabilitation technique that simulates nerve impulses with low-frequency pulsed currents, stimulates muscle contractions, promotes muscle blood circulation, improves muscle nutrient supply, enhances muscle strength and muscle endurance, and regulates neuromuscular excitability to relieve muscle spasms and pain [28]. As the core of the hip abductor muscle group, functional recovery of the gluteus medius plays an important role in maintaining hip stability and improving joint range of motion. The results showed that after 4 weeks of treatment, the NMES group had significantly lower VAS scores, and significantly higher Harris and Berg score than the control group. This finding is consistent with a previous study [29], We speculate that NMES promotes local blood circulation, accelerates inflammatory factor metabolism, and reduces tissue oedema and pain by stimulating gluteus medius contraction. At the same time, regular muscle contraction can prevent muscle fibre atrophy, increase muscle strength, improve hip mechanical balance, and enhance joint function and balance [30].

This study also found that the PSQI score in the NMES group after treatment was significantly lower than that in the control group, indicating that NMES therapy can improve patients’ sleep quality. The correlation analysis further verified the bidirectional vicious cycle between pain and sleep disorders: Before treatment, VAS and PSQI score showed a strong positive correlation in both groups. After treatment, the correlation coefficient decreased in both groups, and the decrease was more significant in the NMES group, changing from a strong positive correlation before treatment to a weak positive correlation, whereas the control group showed only a moderate positive correlation. The mechanism may be that NMES relieves pain by stimulating gluteus medius contraction, reduces nocturnal pain interference with sleep, and helps patients establish a normal sleep rhythm. Improved sleep quality then enhances the body’s anti-inflammatory capacity and tissue repair efficiency, further reducing pain sensitivity, forming a virtuous cycle of “pain relief, sleep improvement, and functional recovery” [31, 32]. A previous study has shown that NMES effectively relieves postoperative pain and promotes early functional recovery by enhancing muscle strength, activating motor units, improving balance and gait stability, which is consistent with the improved Harris hip function scores observed in this study [33]. Additionally, a previous study has also confirmed that postoperative pain is closely associated with sleep disorders [34]; the correlation changes between VAS and PSQI in this study support this conclusion.

This study has several limitations. The sample size is relatively small, which may introduce bias; As a retrospective observational study, it can only reveal correlations and cannot establish a causal relationship. Future prospective cohort studies or randomized controlled trials with mediation/moderation analyses should be conducted to further explore the potential causal pathways and mechanisms between pain and sleep quality. The effect of different electrical stimulation parameters on the treatment outcomes was not examined. Large sample size and multicentre studies are needed to optimize the treatment parameters and further validate the clinical efficacy of NMES.

## Conclusions

NMES applied to the gluteus medius is associated with reduced pain intensity in patients with sleep disorders following HA. This reduction may further contribute to improved sleep quality, thereby alleviating the reciprocal interaction between pain and sleep disorders to a certain extent. Additionally, NMES application is correlated with improvements in hip joint function and balance ability, which may positively affect functional recovery. These findings suggest that NMES is a promising adjuvant rehabilitation approach for patients with sleep disorders after HA.

## Availability of Data and Materials

The datasets used and analysed during the current study were available from the corresponding author on reasonable request.
